# Nonclassical Crystallization of the L‑Tartrate Salt of Cyamemazine

**DOI:** 10.1021/acs.cgd.5c00223

**Published:** 2025-07-15

**Authors:** Sreela Ramesh, Elina Harju, Teemu Tomberg, Jan Rohlíček, Eliška Zmeškalová, Thomas Rades, Clare J. Strachan, Miroslav Šoóš

**Affiliations:** † Department of Chemical Engineering, 52735 University of Chemistry and Technology, Technická 3, Dejvice, 166 28 Prague 6, Czech Republic; ‡ Zentiva, k.s., U.K.abelovny 130, 10237 Prague 10, Czech Republic; § Drug Research Program, Division of Pharmaceutical Chemistry and Technology, Faculty of Pharmacy, 3835 University of Helsinki, Viikinkaari 5 E, 00790 Helsinki, Finland; ∥ Department of Structure Analysis, Institute of Physics of the Czech Academy of Sciences, Cukrovarnická 112/10, 162 00 Praha 6, Czech Republic; ⊥ Department of Pharmacy, Faculty of Health and Medical Sciences, University of Copenhagen, Universitetsparken 2, 2100 Copenhagen, Denmark

## Abstract

This study investigates the nonclassical crystallization of cyamemazine L-tartrate, a kryptoracemic salt of the antipsychotic drug cyamemazine. The crystallization pathway was studied via slurry conversion of a suspension of cyamemazine and L-tartaric acid in 2-propanol to the salt. Notably, the system transitioned through an intermediate droplet phase, which subsequently coalesced to form a dense phase. Both intermediate phases exhibited liquid crystalline character when observed under a polarized light microscope. The droplets demonstrated birefringence and a distinct Maltese cross extinction pattern, confirming their liquid crystalline nature. The dense phase revealed a fan-like texture when isolated and examined between glass slides. Comprehensive analyses of the intermediate droplet phase and the dense phase were conducted using solution ^1^H nuclear magnetic resonance (NMR), X-ray diffraction (XRD), and in situ stimulated Raman scattering (SRS) imaging. The combination of imaging and analytical techniques employed in this study allowed for elucidation of the nature and composition of the intermediate phases involved in the crystallization process.

## Graphical abstract

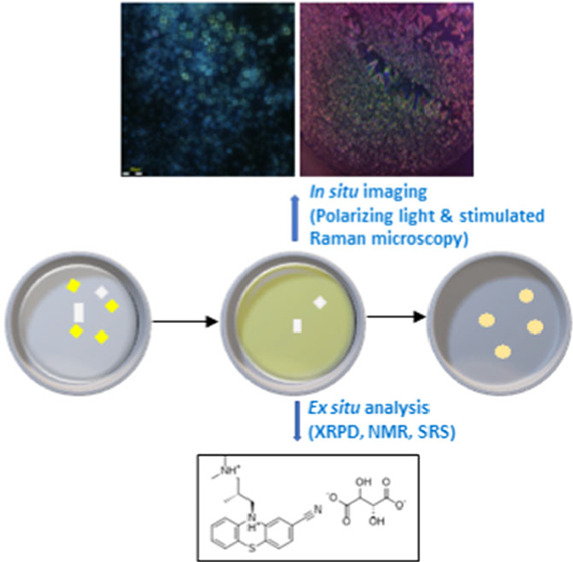

## 1 Introduction

Cyamemazine is an antipsychotic drug belonging to the class of phenothiazines and is marketed as either the free base or its L-tartrate salt under the brand name Tercian.^1^ It is amphiphilic and has an asymmetric carbon atom in its structure but is marketed in the racemic form. It has a tertiary amine group susceptible to protonation, which makes the formation of salts with acids possible. Our previous study^2^ described novel salts of cyamemazine with dicarboxylic acids and their crystal structures, from which it was evident that the marketed L-tartrate salt is a kryptoracemate.^3^ Kryptoracemates are rare types of racemic crystals without an inversion center where both enantiomers are present in the asymmetric unit. In the salt formation between cyamemazine and L-tartaric acid (Figure 1), the basic amine group of cyamemazine interacts with one of the carboxylic acid groups of L-tartaric acid, resulting in the formation of a charge-assisted hydrogen bond, while the second carboxylic acid group remains unreacted.

**Figure 1:**
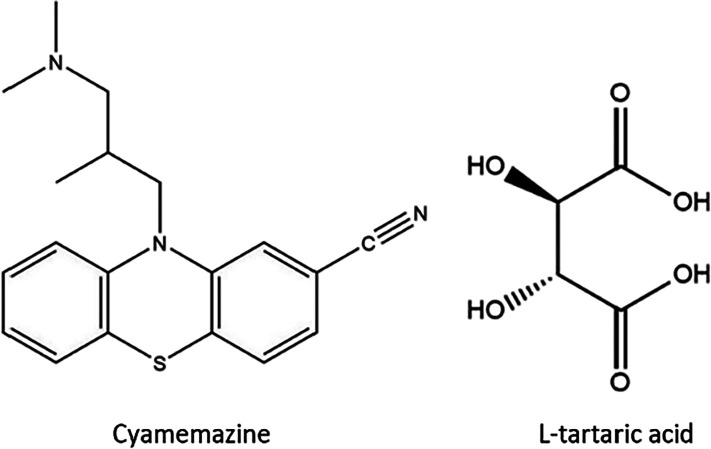
Chemical structures of cyamemazine and L-tartaric acid.

Nonclassical crystallization differs from the classical crystallization mechanism, in that it involves building units (nanoparticles, droplets, clusters, etc.) and not a layer-by-layer crystal growth from atomic or molecular building units.^4^ A combination of various nonclassical pathways is also possible, where nanodroplets might transform to amorphous nanoparticles^5^ or initial amorphous spherical agglomerates might densify and, for example, produce a crystalline order first, from which crystal growth occurs.^6^ Very few examples of nonclassical crystallization in pharmaceutical materials have been reported so far. Warzecha et al.^7^ reported that the nonclassical crystallization of olanzapine dihydrate occurs in two steps and described how crystallization conditions affect the final form. Under unstirred conditions, initially nanodroplets are formed that are then transformed to the thermodynamically stable crystalline form (a dihydrate) by templating. In contrast, stirring leads to detachments of droplets and, hence, influences the kinetics of dihydrate formation. Another example of multistep crystallization in pharmaceuticals is a report on the evolution of amorphous cyclosporin A nanoparticles to single nanocrystals via oriented attachments of polycrystalline aggregates.^8^ The role of liquid phase precursors in the formation of ibuprofen has also been reported.^9,10^ Flufenamic acid, a nonsteroidal anti-inflammatory drug, has been found to form via prenucleation clusters,^11^ which then coalesce to form a densified intermediate material from which nucleation formation occurs.

In order to understand the nature of the intermediate phases in nonclassical crystallization processes, in situ imaging and other analytical methods are necessary. Direct microscopic observations using liquid-phase electron microscopy,^11,12^ cryogenic transmission electron microscopy (cryo-TEM),^6,13,14^ and atomic force microscopy^14^ have been employed to probe such intermediate phases in recent years, whereas coherent Raman techniques can be used for 2D- and 3D-imaging of component distribution.^15^

Liquid crystals, a state of matter between the liquid and crystalline (solid) state, have been reported to appear during crystallization processes.^16,17^ Liquid crystalline droplets that appear as intermediates^18,19^ have mostly been described in nonclassical crystallization of biomolecules from solutions. Even though there are numerous applications of liquid crystals in pharmaceutics,^20,21^ there are only few reports on the role of liquid crystal droplet intermediates in the crystallization processes of low molecular weight drugs. Cyamemazine is so far not known to form liquid crystals but lyotropic liquid crystals of chlorpromazine hydrochloride, a closely related drug, have been discovered.^22,23^

This study focuses on imaging of the nonclassical crystallization process involved in the formation of cyamemazine L-tartrate salt from isopropanol. Utilizing polarizing light microscopy and in situ stimulated Raman scattering (SRS) imaging, we elucidate the different steps of the crystallization pathway. We further characterized the intermediate phases through solution ^1^H nuclear magnetic resonance (NMR) and X-ray diffraction (XRD) analyses. The integration of these imaging and analytical techniques provides comprehensive insights into the crystallization dynamics and the properties of the resulting cyamemazine L-tartrate salt.

## 2 Experimental Section

### 2.1 Materials

Cyamemazine and its L-tartrate salt (used as reference material) were kindly provided by Zentiva, k.s. (Prague, Czech Republic). Isopropanol and L-tartaric acid were of analytical purity and were obtained from Sigma-Aldrich and used as received.

### 2.2 Preparation of the L-Tartrate Salt

Cyamemazine (10 mg) at a 1:1 molar ratio with L-tartaric acid (4.64 mg) was added to 2 mL of isopropanol in a 35 mm glass bottom cell culture dish used as a crystallization pan and kept closed to avoid evaporation of solvent. The transformation of the drug to its L-tartrate salt thus happened through slurry conversion.

### 2.3 Analytical Methods

#### 2.3.1 XRD

Cyamemazine and L-tartaric acid (in a 1:1 ratio) were ground and placed in a 0.5 mm borosilicate-glass capillary with a diameter of 1 mm on each end. Isopropanol was added to the capillary, as much as possible without disturbing the solids, before the addition of L-tartaric acid crystals but after cyamemazine was added to the capillary. Only the middle of the capillary was irradiated, where no signal from unreacted starting materials was expected and 42 powder diffraction patterns were collected, each measured for 1 h. Powder diffraction data were collected using the Debye–Scherrer transmission configuration on the powder diffractometer Empyrean PANalytical (Malvern Panalytical, Almelo, Netherlands, λCu,Kα = 1.54184 Å, voltage 45 kV and operating current 40 mA) that was equipped with a focusing mirror, capillary holder, and PIXcel3D detector. A capillary made from a borosilicate glass was used during the in situ experiments. The range of the measurement was 5–30 ° 2θ, the step size was 0.013 ° 2θ, and the measurement rate was 150 s per step.

#### 2.3.2 Optical Microscopy

Samples were viewed using a DSX1000 digital microscope (Olympus, Tokyo, Japan) using dark field and polarized light (the latter to detect the birefringence of the liquid crystalline and crystalline phases). The samples prepared in the crystallization pans were viewed for in situ tracking of the crystallization process. The dense phase was isolated, sandwiched between two glass slides, and imaged as soon as possible under polarized light. The images of the samples were recorded at room temperature (25.0 °C).

#### 2.3.3 Solution ^1^H NMR Spectroscopy

Samples were dissolved in d6-DMSO, and ^1^H NMR spectra were measured using a Bruker Avance III 500 MHz NMR spectrometer (Bruker Biospin AG, Faellanden, Switzerland) equipped with a Prodigy probe (5 mm) and a repetition delay of 10 s. Chemical shifts (δ) are reported in ppm downfield from tetramethylsilane. Spectra were calibrated against the residual solvent peak from DMSO (at 2.50 ppm) and were analyzed in Topspin software 3.2.

#### 2.3.4 Spontaneous Raman Spectroscopy

Samples for Raman spectroscopy were measured in HPLC glass vials in an FT-Raman RFS100/S spectrometer (Bruker Optics, Bremen, Germany), equipped with a Germanium detector. The wavelength of the Nd:YAG laser was 1064 nm. The measuring range was from 4000 to 200 cm^–1^, with a spectral resolution of 4 cm^–1^. Data were obtained at 64 accumulations of the measured spectra. OMNIC (Thermofisher Scientific) and OPUS (Bruker) software programs were used to record the Raman spectra.

#### 2.3.5 SRS Microscopy

SRS microscopy was performed using a customized Olympus FV3000 confocal laser scanning microscope (Olympus, Tokyo, Japan).^24^ The SRS signal was collected in the transmitted direction. Reference and sample spectra were recorded in situ between 2195 and 2264 cm^–1^ with a 3 cm^–1^ step size and a pump laser wavelength of 850 nm. The SRS images were preprocessed by min-max normalization, block-matching, and 4D filtering (BM4D) for noise reduction and baseline adjustment through minimum value subtraction, using an in-house developed MATLAB-based application.

## 3 Results and Discussion

### 3.1 Visual Appearance of the Crystallization Process

Prior to imaging the crystallization process with microscopy, the formation of the L-tartrate salt from a suspension of cyamemazine and L-tartaric acid in isopropyl alcohol was observed with the naked eye. This preliminary observation aimed to identify the constituents present in the crystallization pan based on their morphological characteristics, facilitating more targeted investigations in subsequent experiments. At room temperature, most cyamemazine crystals dissolved rapidly in the solvent without the application of stirring or heating, while L-tartaric acid crystals displayed limited solubility. Visual observations revealed that as cyamemazine dissolved, the solution transitioned to a yellowish hue with transparent L-tartaric acid crystals remaining suspended. Within the first 2 min of the experiment, yellow-colored “streams” (Figure S1) became visible, moving among the undissolved acid crystals. The solution increased in viscosity, while simultaneously giving way to the formation of yellow spherical agglomerates, identifiable as salt spherulites. At the conclusion of the crystallization process, the presence of solely the salt in the pan was observed after 24 h, with minimal to no solvent remaining, likely due to evaporation over an extended period, despite the pan being closed. In situ*-*polarized light microscopy imaging of this crystallization process was performed next and was anticipated to provide insights into the nature of the yellow streams formed initially and the reason for the increased viscosity afterwards.

### 3.2 Liquid Crystalline Character of Intermediate Phases

The crystallization process was imaged under polarized light to investigate the formation of crystal nuclei and intermediate phases. In Figure 2, microscopy images are shown alongside a sketch of the steps in this process. As crystallization progresses, droplets [Figure 2a] with diameters between 5 and 10 μm appear in the solution, which then move between the undissolved acid crystals (SI Video 1 and Figure S2). When viewed under polarized light, the droplets display birefringent Maltese cross extinction patterns (Figure 3), which suggest a liquid crystalline nature. These unique patterns are usually observed in droplets when they have a radial director configuration.^25^

**Figure 2:**
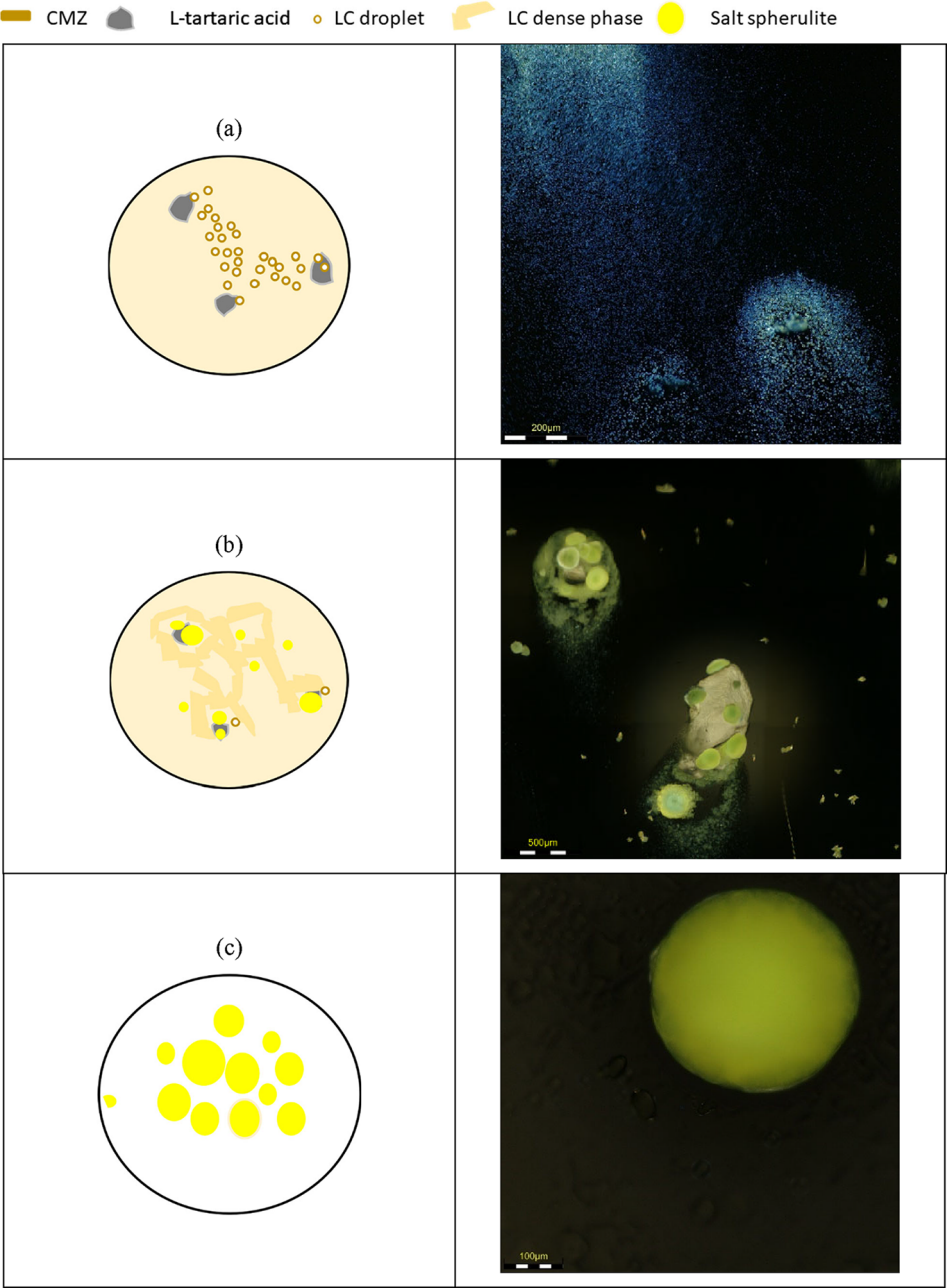
Polarizing light microscopy images of (a) droplets moving between and gathering around acid crystals, (b) salt spherulites forming on top of acid crystals, and (c) fully grown L-tartrate salt spherulite.

**Figure 3:**
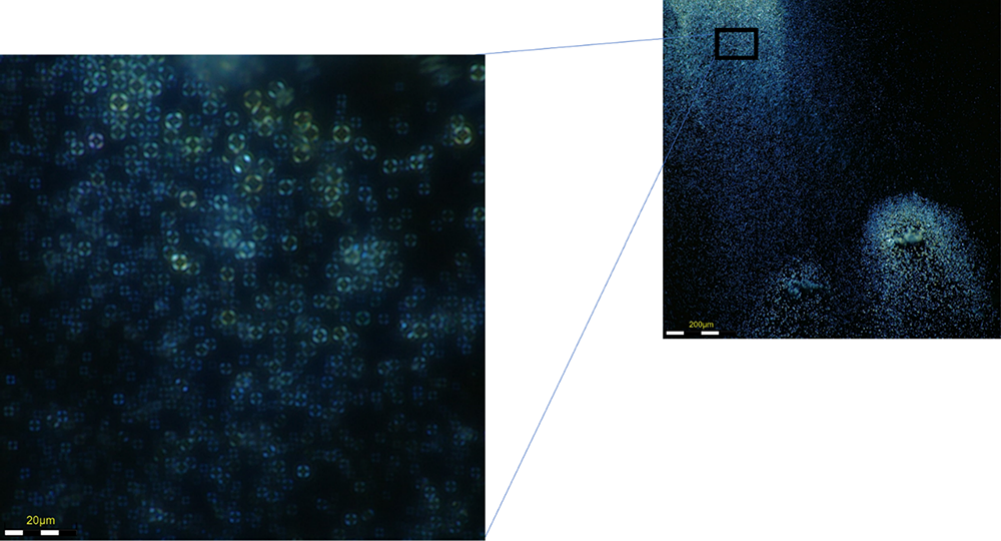
Maltese cross-extinction patterns observed in the intermediate droplet state were obtained using polarizing light microscopy.

Since the droplets are expected to possess a radial director configuration (Figure 4) and exhibit self-propulsion within the solution, they can be classified as active droplets.^26^ The internal anisotropy introduced by the radial alignment, together with the emergence of spontaneous concentration gradients, breaks the initial isotropic conditions of the system.^27,28^ Movement is, hence, expected to be driven by Marangoni effects, which arise from surface tension gradients caused by the spatial variation in reactant concentration within the suspension.^29^ Additionally, the ongoing chemical reaction that favors salt formation further contributes to the droplet’s propulsion, facilitating their dynamic behavior in the medium.

**Figure 4:**
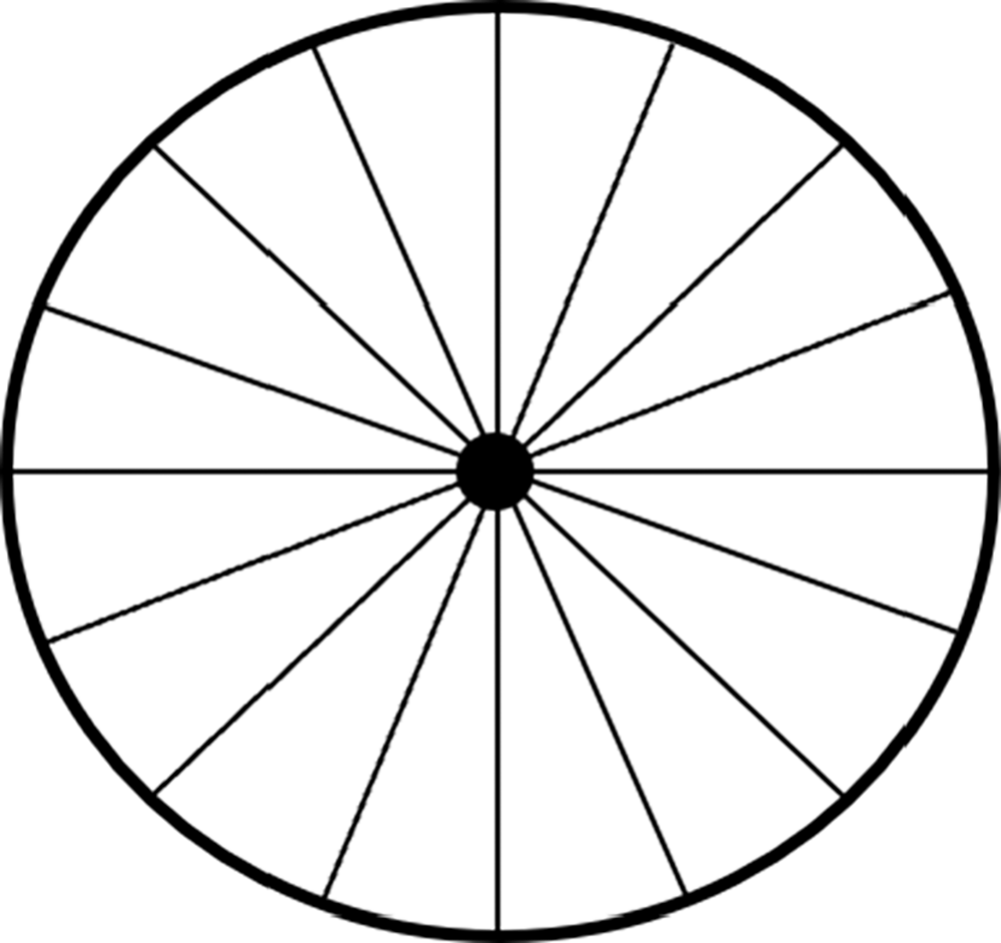
Radial director in the droplets.

Droplets accumulate near L-tartaric acid crystals and subsequently coalesce, forming a dense phase, as depicted in Figure 2b. Simultaneously, salt spherulites begin to grow on the surface of the acid crystals and expand in size at the expense of the surrounding dense phase (SI Video 2). Although droplet formation stands out as a separate and initial event, the subsequent stages of crystallization happen simultaneously. A higher frequency of droplet formation is observed in the vicinity of larger acid crystals or regions with a higher density of acid particles (Figure S3). Upon completion of the crystallization process, the entire pan is populated by spherulites. The fully developed spherulites, as shown in Figure 2c, have an average diameter of approximately 300–500 μm.

When the dense phase is isolated and sandwiched between two glass slides for observation under polarized light, fan-like textures are observed (Figure 5a,b), indicating the liquid crystalline nature of this phase. Imaging of the dense phase was performed immediately after isolation to prevent or minimize changes in solvent composition. Upon complete drying on the glass slide, the dense phase remained as a sticky yellow substance, retaining its fan-like texture.

**Figure 5:**
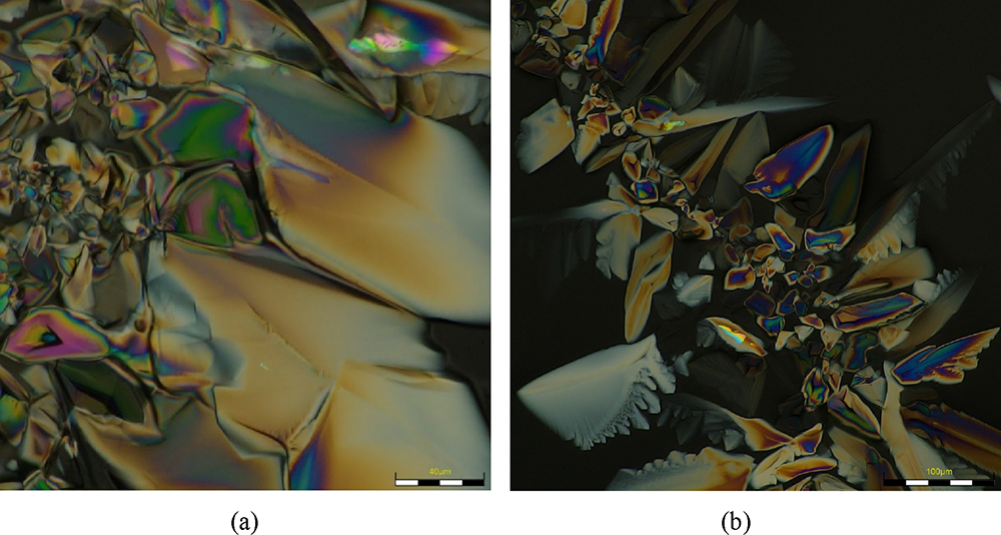
(a, b) Fan-like textures observed when the dense phase (formed by coalescence of droplets) is sandwiched between glass slides and analyzed under polarized light.

Given that the crystallization process proceeds via a nonclassical mechanism, the possibility of polymorphs of L-tartrate salt occurring, aside from the kryptoracemic form,^30^ cannot be disregarded. The properties of the precursor formed during crystallization could significantly influence the nucleation of specific polymorphic forms. Previous studies have highlighted the role of solute–solute interactions in the early stages of nucleation, or even prior to it, in promoting the formation of structures with *Z*′ > 1, where *Z*′ denotes the number of formula units in the asymmetric unit.^31^ Kryptoracemates, such as the L-tartrate salt studied here, fall into this class of structures. These structures are often found to be metastable, suggesting that kryptoracemates may represent metastable or kinetic products of crystallization. This implies that more stable polymorphic forms could exist but remain hidden unless favorable conditions promote their appearance.

To date, no polymorphs other than the kryptoracemic form of cyamemazine L-tartrate have been reported. It was, therefore, essential to monitor for the emergence of polymorphic or other transient solid forms during the nonclassical crystallization process described in this study. XRD and NMR measurements were employed to detect the presence of such forms, and these experiments were conducted next.

### 3.3 Polymorph Characterization Using XRD

To replicate the experimental conditions in the crystallization pan as closely as possible during XRD experiments, a capillary was used to conduct the slurry conversion of cyamemazine and L-tartaric acid into the L-tartrate salt of cyamemazine. In situ XRD patterns were recorded throughout the process with the aim of identifying any solid forms that emerge during the crystallization.

Figure 6a presents a range of X-ray diffraction patterns between 10° and 20° 2θ, where three measurements are summed to enhance the visibility of the diffraction peaks, while Figure 6b illustrates the capillary setup during measurement, showing the formation of L-tartrate salt in the irradiated area, while undissolved starting materials, cyamemazine and L-tartaric acid, remain visible at opposite ends. In Figure 6c, the last measured XRD pattern of the sample, presented without background interference, is compared with reference patterns for cyamemazine, L-tartaric acid, and kryptoracemic L-tartrate salt. Notably, from Figure 6a,c, no new or unknown XRD patterns were detected during the measurements, indicating the absence of any other polymorphic forms of L-tartrate salt crystallizing from the intermediate droplet and dense phases.

**Figure 6:**
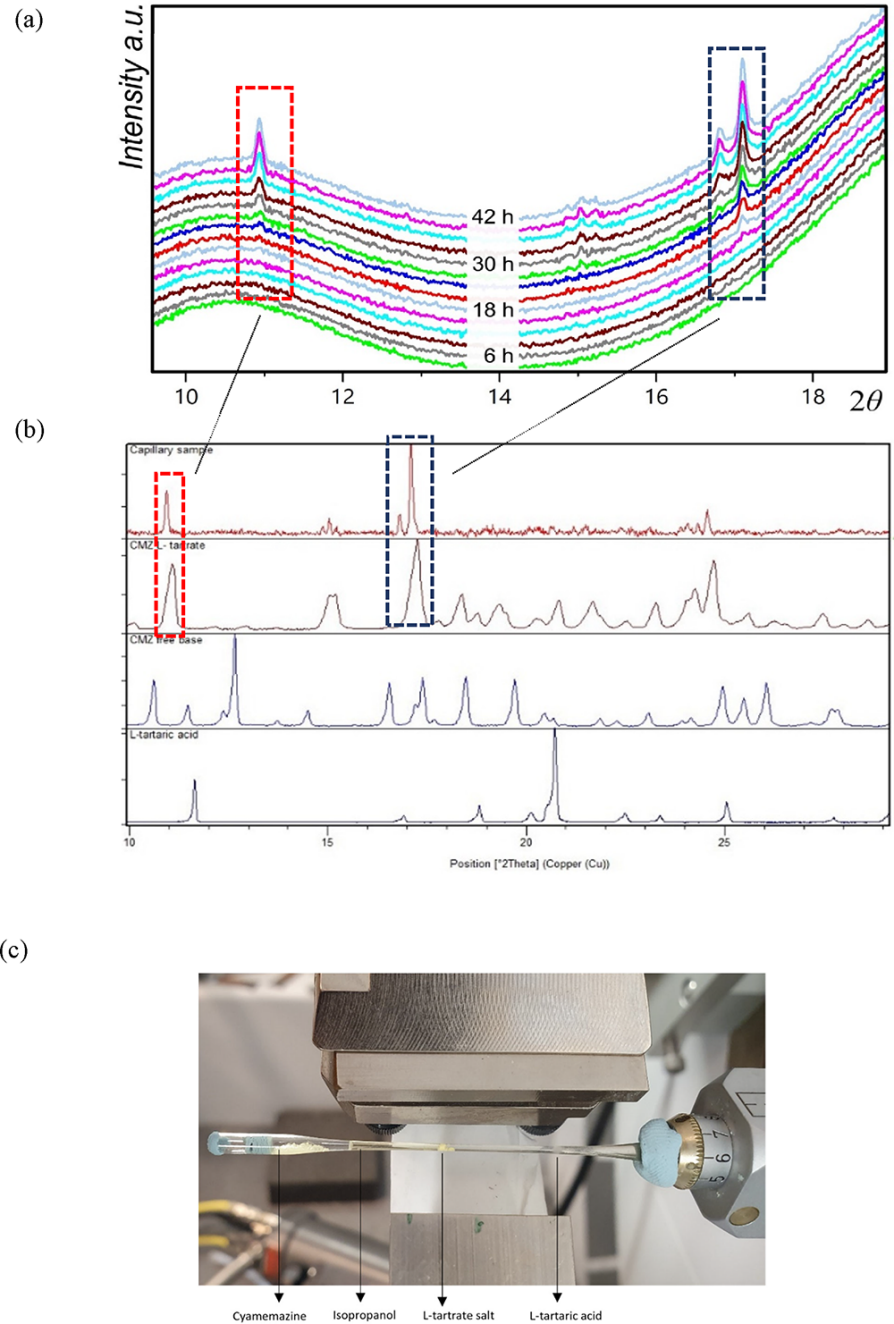
(a) XRD patterns of the capillary, where conversion of reactants to the salt occurs. Every pattern in this graph is a summation of three consecutive 1 h measurements. The high background is caused by the glass walls of the capillary and also by the presence of the liquid isopropanol in the capillary. (b) Comparison of the last XRD pattern of the sample formed in the middle of the capillary without a background to the starting materials and expected product. (c) Photo of the capillary setup taken during the measurement with all components of the reaction system marked.

### 3.4 Analysis of the Dense Phase Using Solution ^1^H NMR

The dense phase that occurs during the crystallization process is more long-lived and stationary in the crystallization pan compared to the droplet phase. Upon probing the pan with a spatula, it was observed at the bottom of the pan as a yellow sticky phase. A portion of this dense phase was isolated from the reaction mixture and characterized using solution NMR (Figure 7). A comparison of the NMR spectrum of this sample with that of the reference cyamemazine L-tartrate salt showed that the dense phase is indeed composed of the salt. Consistent with the results obtained from XRD measurements, this finding reinforces the hypothesis that no additional solid forms were present during the intermediate phases of the reaction. The presence of a small amount of 2-propanol in the sample was unavoidable during the isolation of the dense phase. Given that the measurement was conducted shortly after isolation, the likelihood of complete evaporation of 2-propanol from the sample is low. Consequently, peaks attributed to 2-propanol were detected in the NMR spectrum of the sample. The sharp peaks at 1.1 and 3.8 ppm correspond to the CH hydrogens in isopropanol, while the broader peaks may be associated with hydrogen groups from both isopropanol and water.

**Figure 7:**
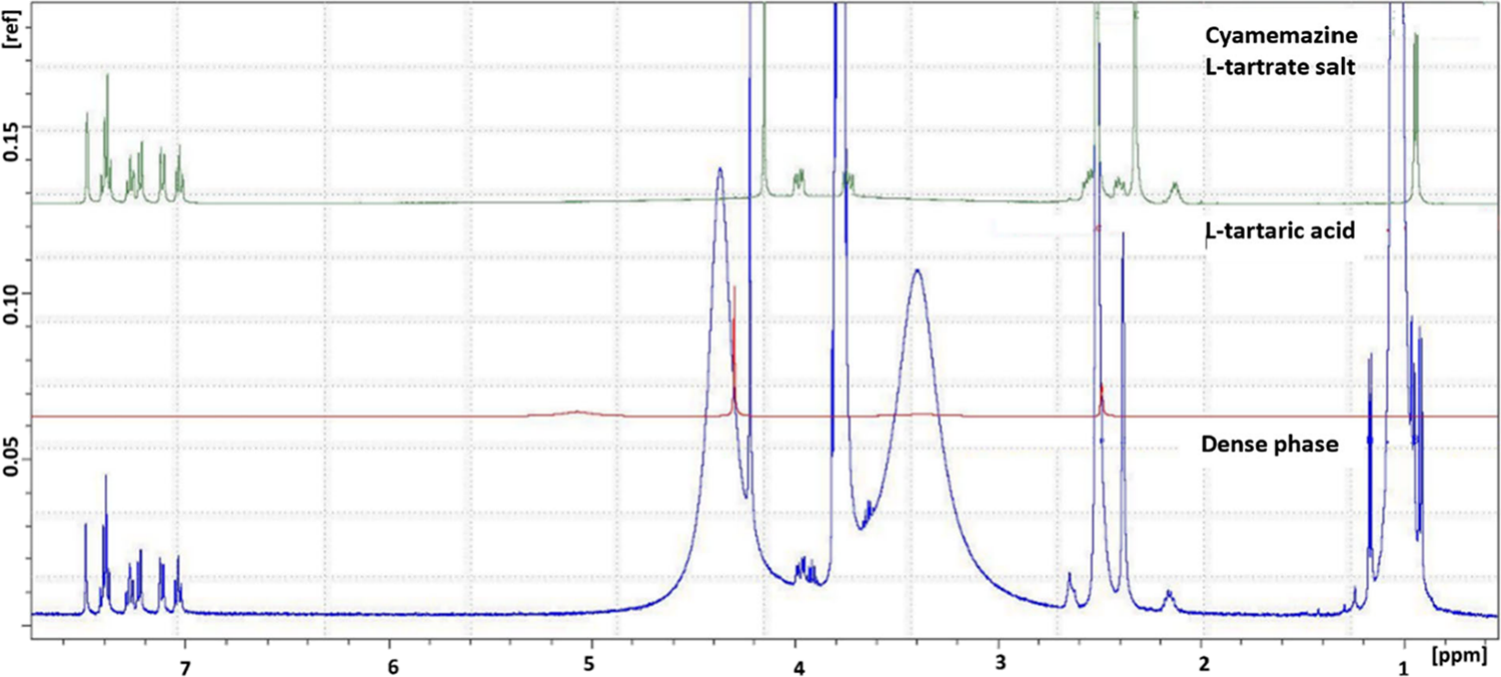
Solution ^1^H NMR spectrum of the dense phase isolated from the crystallization slurry compared to those of L-tartaric acid and L-tartrate salt.

### 3.5 In Situ SRS Imaging and Characterization

SRS microscopy not only facilitated high-resolution in situ imaging of the crystallization process but also enabled the characterization of intermediate phases within a specific Raman spectral region. Since the methods employed previously for analysis of the dense phase could not be used for the droplet phase due to its transient nature, SRS microscopy is expected to be a vital tool for chemical characterization of the droplets. It also serves as a reliable technique to confirm that the dense phase comprises the L-tartrate salt.

Given that both cyamemazine and the L-tartrate salt contain a nitrile group functionality, the optimal spectral range for probing was identified as the Raman shift region of 2195–2264 cm^–1^, which corresponds to the nitrile stretching vibrations of the cyamemazine molecule in both of these compounds. The formation of a hydrogen bond between nitrile group and L-tartaric acid in the salt is a plausible explanation for this observed shift. Moreover, nitrile vibrations in general are well-known for being sensitive to hydrogen bonding, and the direction of the shift (blue or red) depends on the nature and geometry of the interaction.^32,33^

In contrast, other spectral regions of cyamemazine and the L-tartrate salt either showed insufficient differentiation for discriminatory analysis or exhibited strong Raman signals from background sources. In order to obtain the reference SRS spectra, hyperspectral images of cyamemazine and L-tartrate salt were recorded separately, as shown in Figure 8a. Upon comparison of the Raman shift [Figure 8a] corresponding to the nitrile stretch vibration of the reference materials, a 3 cm^–1^ difference was observed between the Raman peak center positions. The nitrile functionality in cyamemazine displayed a Raman shift at 2226 cm^–1^, while the vibration of the same functionality in the L-tartrate salt appeared at a shift of 2229 cm^–1^. Furthermore, to confirm the observed peak position difference, FT-Raman measurements were conducted to record the spectra of both cyamemazine and L-tartrate salts [Figure 8b]. The 3 cm^–1^ difference in Raman peak positions was observed in the FT-Raman spectra, as well. Since the observations from SRS and FT-Raman measurements were in agreement, the nitrile stretch region of the spectra was ultimately chosen to proceed with in situ imaging.

**Figure 8:**
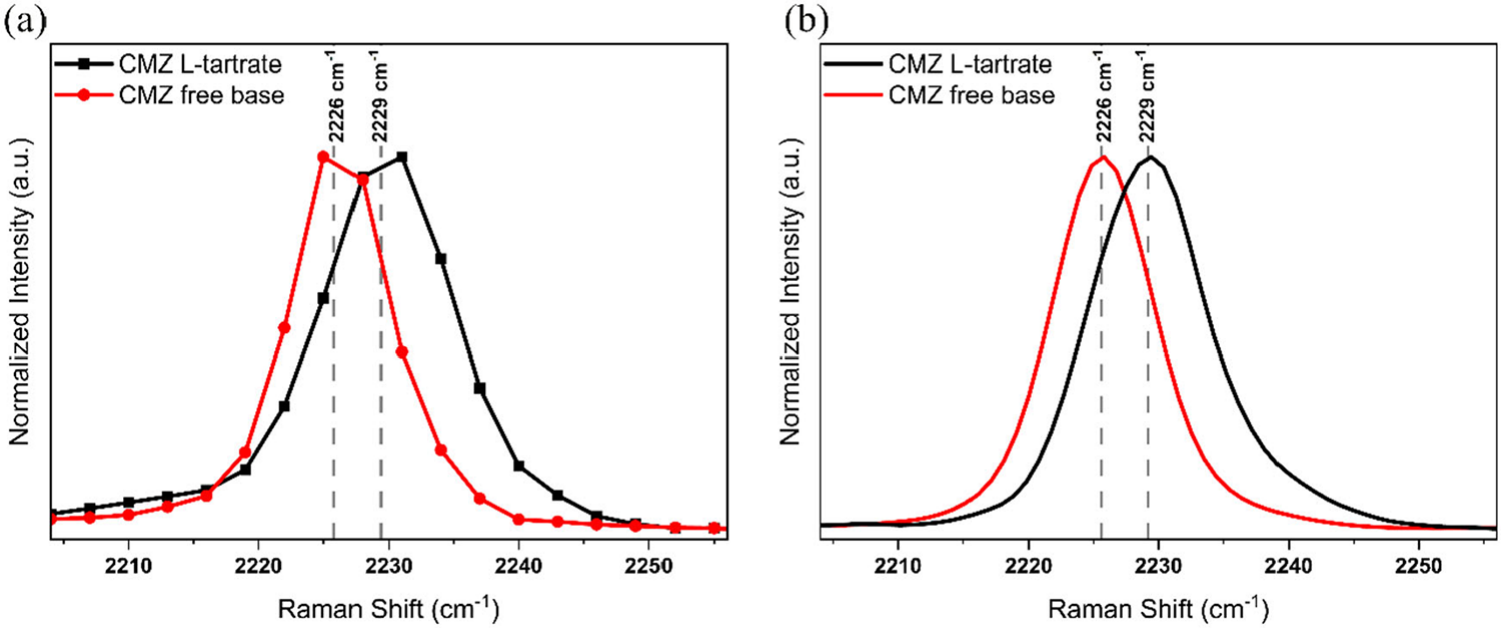
(a) SRS spectra of cyamemazine and L-tartrate salt in the nitrile stretch region of the cyamemazine molecule. (b) FT-Raman spectra of cyamemazine and L-tartrate salt.

SRS spectra from crystals, droplets, and dense phase in the crystallization pan were expected to provide structural information about them in real-time. Even though it is speculated from XRD and NMR analyses that the dense phase is composed of the kryptoracemic L-tartrate salt, it would be helpful to have further confirmation through SRS in situ imaging. Since the crystallization process has been observed before using polarized light microscopy, it was possible, at this point in the study, to identify the different stages and components in the crystallization pan from their morphologies. Cyamemazine crystals are visible as yellow blocks, droplets are spherical and always on the move, L-tartaric acid crystals are transparent and bigger in size compared to cyamemazine, the dense phase has an irregular shape, and the final product, L-tartrate salt, occurs in the form of yellow spherulites.

As the crystallization progressed, droplets started to form, and an image of this part of the crystallization pan [Figure 9a] representing the SRS signal intensity at 2228 cm^–1^ was captured. Both cyamemazine and the L-tartrate salt were expected to exhibit the SRS signal (though not their maxima) at this intermediate Raman shift. Morphologically, a cyamemazine crystal and droplets were identified. Examination of the extracted spectra supported this observation, with the crystal exhibiting a Raman peak at 2226 cm^–1^ consistent with the crystalline cyamemazine reference spectrum and the droplets exhibiting a Raman peak at 2229 cm^–1^, corresponding to the reference salt form of L-tartrate. The CLS analysis [Figure 9b] also classified the crystal-like particle as consisting of cyamemazine and the droplets as consisting of the L-tartrate salt. Figure 9c represents the Raman spectra from the region of interest (ROIs) marked in Figure 9a, in which the different shifts are visible.

**Figure 9:**
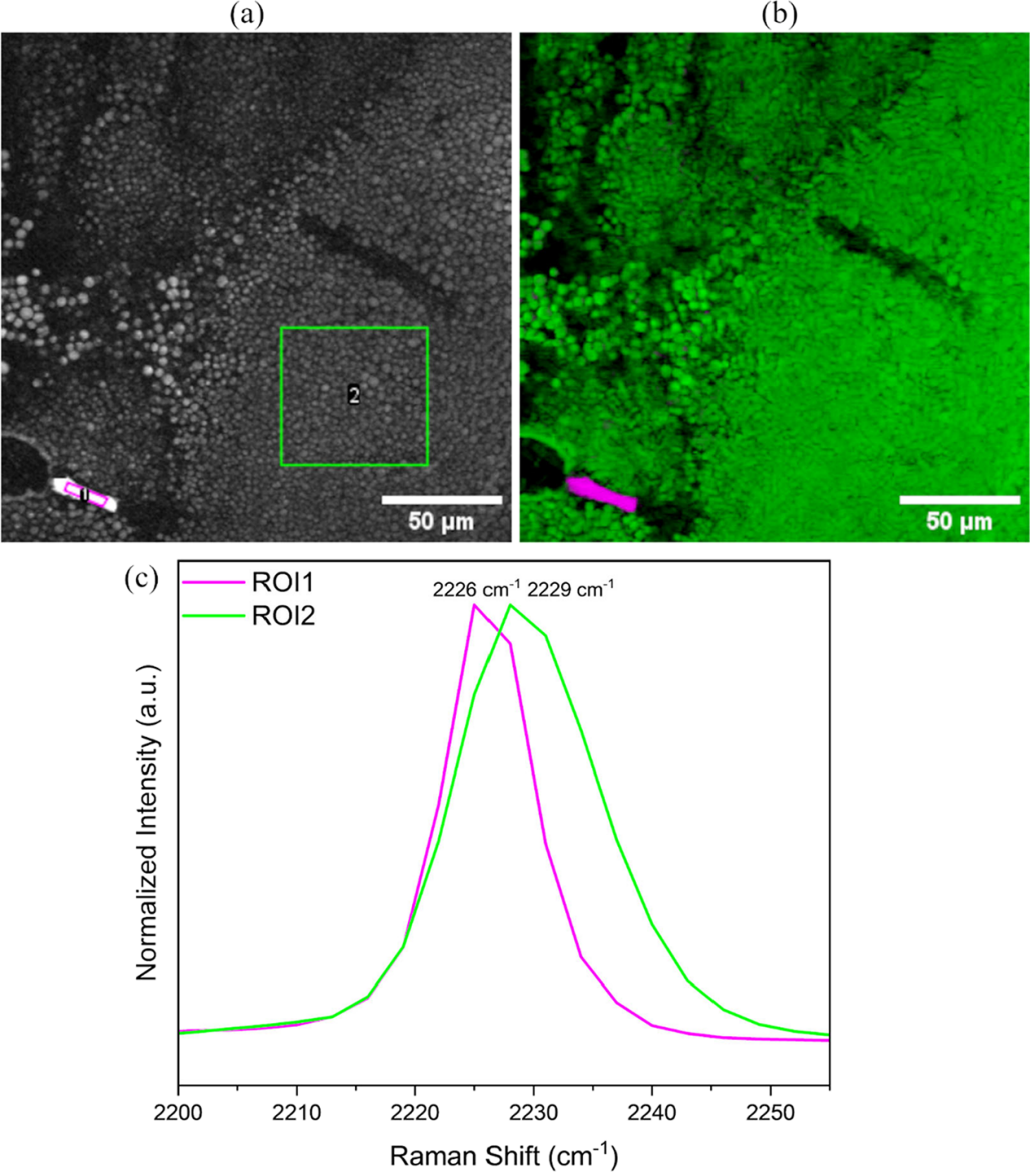
(a) SRS image at 2228 cm^–1^ showing coexistence of intermediate droplet phase and cyamemazine; (b) CLS analysis for which reference spectra of cyamemazine and L-tartrate salt were used as inputs, green: intermediate droplets, magenta: cyamemazine, (c) Raman spectra from the ROIs selected in (a).

As the crystallization progressed, the droplets, identified using SRS analysis as the L-tartrate salt, coalesced to form a dense liquid crystalline phase near the acid crystals, as was also observed when the crystallization process was imaged using polarized light. At this point, salt spherulites had also started to appear, and imaging via SRS microscopy indicated their presence on the surface of acid crystals as growing spherulites. This was also observed when polarized light was used, and it is described in Section 3.2. Inferences from XRD and NMR measurements suggest that the dense phase is composed of L-tartrate salt, and an SRS spectrum recorded in situ can confirm this. A part of the crystallization pan where both the liquid crystalline dense phase and growing salt spherulites were present was identified based on morphologies. An image representing the SRS signal intensity from this part of the pan at 2228 cm^–1^ was captured [Figure 10a]. Examination of the extracted spectra showed that the dense phase, indeed, had a Raman peak at 2229 cm^–1^ corresponding to that of the L-tartrate salt. The salt spherulites also exhibited the same Raman peak, which was also expected. A small undissolved crystal exhibiting a Raman peak at 2226 cm^–1^ was also identified from the image. We expect this to be an undissolved cyamemazine crystal, which is visible only under high-resolution SRS imaging because upon observation using the naked eye and under polarized light, the presence of cyamemazine was not detected near the dense phase or salt spherulites. The CLS analysis [Figure 10b] also classified the small crystal-like particle as consisting of cyamemazine and the dense phase and salt spherulites as consisting of the L-tartrate salt.

**Figure 10:**
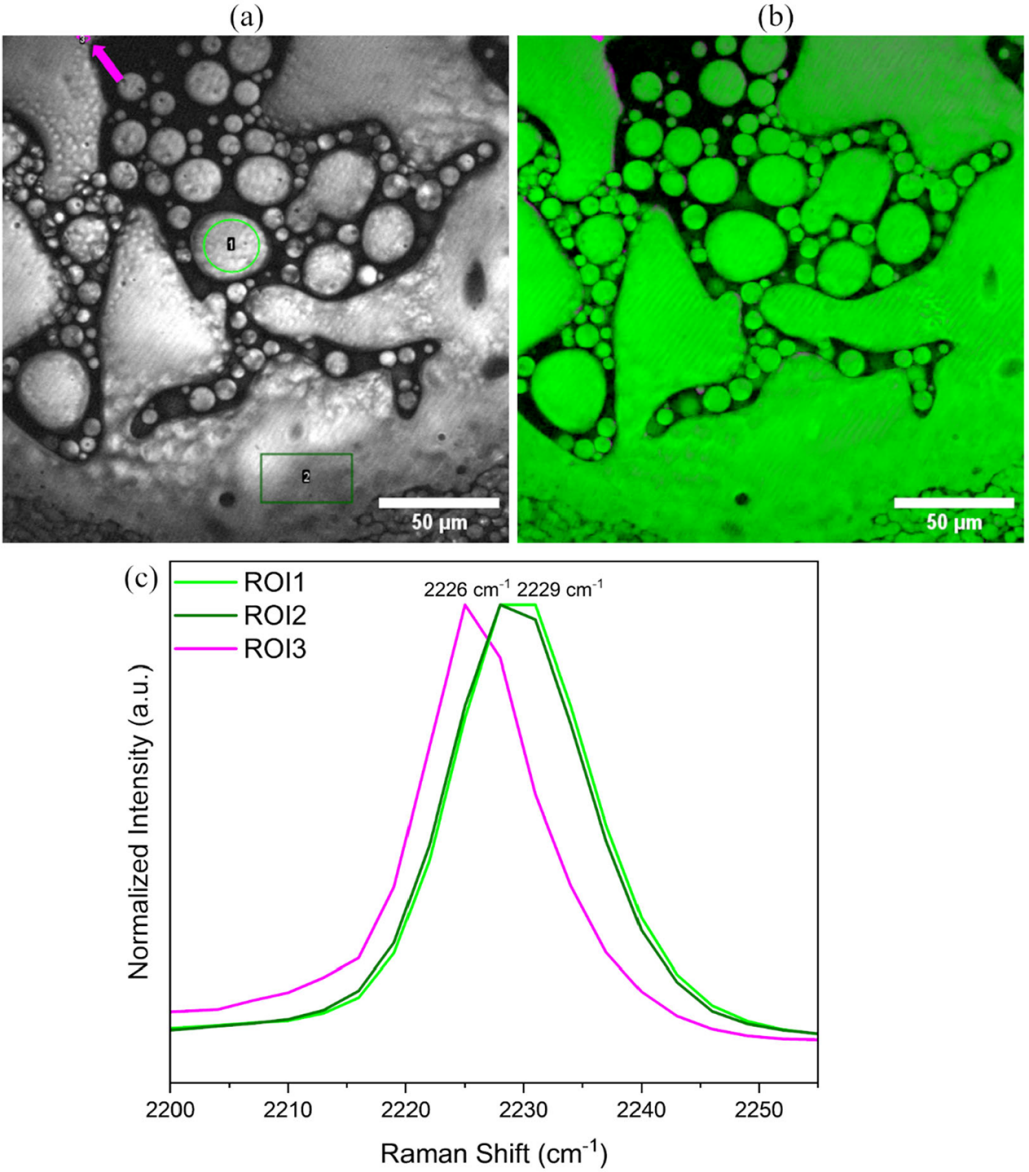
(a) SRS image at 2228 cm^–1^ showing coexistence of intermediate dense phase (irregularly shaped) and L-tartrate salt spherulites (circular); (b) CLS analysis where reference spectra of cyamemazine and L-tartrate salt were used as inputs; green: dense phase and L-tartrate salt; (c) Raman spectra from the ROIs selected in (a).

These results not only confirm the presence of a nonclassical mechanism in the crystallization of the L-tartrate salt but also demonstrate that both the liquid crystalline droplets and the dense intermediate phases are composed of the L-tartrate salt.

### 3.6 Overview of the Crystallization Mechanism

The crystallization of cyamemazine L-tartrate salt from isopropanol follows a nonclassical pathway, as demonstrated through the various studies outlined in this article. Figure 11 presents a comprehensive overview of the various steps involved in this pathway.

**Figure 11:**
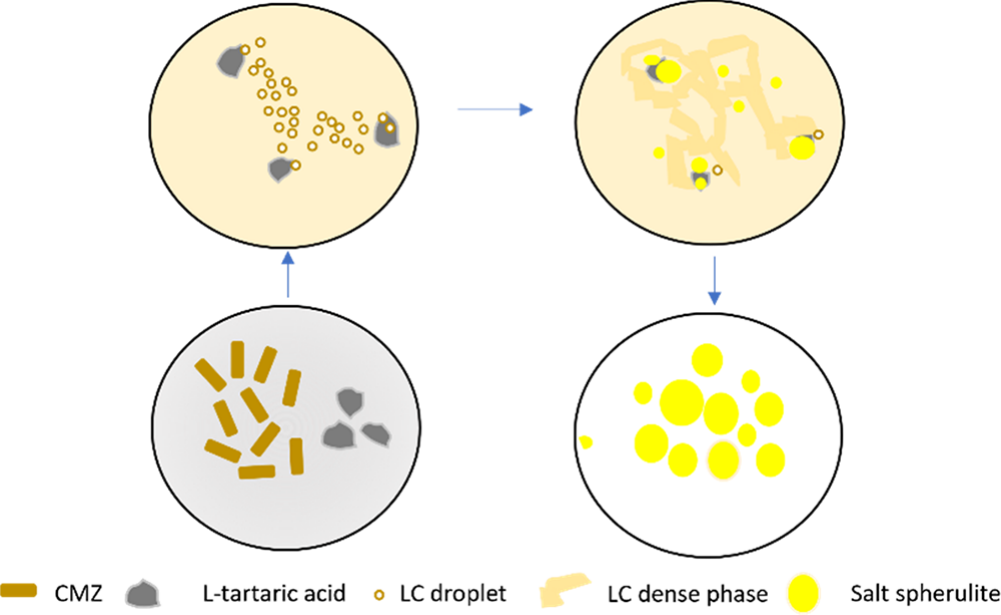
Overview of the nonclassical crystallization process of cyamemazine L-tartrate.

Upon suspension of cyamemazine and L-tartaric acid crystals in isopropanol, cyamemazine predominantly dissolves, initiating the formation of LC droplets containing the L-tartrate salt. These droplets exhibit mobility toward the L-tartaric acid crystals, driven by the Marangoni effect. Subsequently, the LC droplets coalesce, forming a dense LC phase enriched with the L-tartrate salt in the proximity of the acid crystals. Crystallization of L-tartrate salt occurs as spherulitic structures on the surface of the L-tartaric acid crystals, with these spherulites growing at the expense of the dense LC phase, while the L-tartaric acid is simultaneously consumed by the reaction. The involvement of an intermediate LC droplet state is critical to advancing the reaction, which would not proceed under classical crystallization conditions, as L-tartaric acid is insoluble in isopropanol. These findings suggest that the nonclassical crystallization mechanism, characterized by the formation of LC phases and intermediate droplets, may be a preferred pathway for the crystallization of cyamemazine salts, particularly in systems where the compounds exhibit metastability or favor kinetic products. Further investigation into the crystallization behavior of other kryptoracemic salts of cyamemazine is required to determine whether this nonclassical pathway is a general feature of this class of compounds. The influence of solvent selection on the crystallization mechanism also remains to be studied extensively to gain a complete understanding of the salt formation process.

## 4 Conclusions

In this study, the crystallization mechanism of the marketed cyamemazine L-tartrate salt, which is a kryptoracemate, was investigated using in situ microscopic techniques, NMR and XRD. Crystallization from a suspension of cyamemazine and L-tartaric acid in isopropanol adopts a nonclassical pathway, forming intermediate droplets, which then coalesce to form a dense phase. Polarizing optical microscopy suggests a liquid crystalline nature of both the intermediate droplet and the dense phases involved in the crystallization. Both these phases are composed of the L-tartrate salt, and no other solid form or polymorph of L-tartrate salt was detected throughout the process.

## Supplementary Material







## Notes

Yellow-colored streams of droplets, polarizing light microscopy image of droplets moving toward acid crystal, dark field microscopy image of crystallization pan, XRD of cyamemazine and L-tartrate salt in isopropanol measured while placed in a capillary compared to their powder XRD patterns, PCA of SRS microscopy images, and SRS images of cyamemazine and L-tartrate salt (PDF)
Video of droplets moving in the crystallization pan (MP4)
Video of salt spherulites growing at the expense of dense phase (MP4)
