# Supplementary material for: Nonclassical Crystallization of the L‑Tartrate Salt of Cyamemazine
Source: Cryst Growth Des. 2025 Jul 15;25(15):5813–21. doi: 10.1021/acs.cgd.5c00223 (PMC12333587; doi:10.1021/acs.cgd.5c00223)
Supplement: Supplementary file 1 [file cg5c00223_si_001.pdf]

Supplementary material

Non-classical crystallization of the L-tartrate salt of cyamemazine

Sreela Ramesh[1,2], Elina Harju[3], Teemu Tomberg[3], Jan Rohlíček[4], Eliška Zmeškalová[1,4], Thomas Rades[5], Clare J. Strachan[3], Miroslav Šoóš[1*]

[1] Department of Chemical Engineering, University of Chemistry and Technology, Technická 3, 166 28 Prague 6 – Dejvice, Czech Republic

[2] Zentiva, k.s., U Kabelovny 130, 10237 Prague 10, Czech Republic

[3] Drug Research Program, Division of Pharmaceutical Chemistry and Technology, Faculty of Pharmacy, University of Helsinki, Viikinkaari 5 E, 00790 Helsinki, Finland

[4] Department of Structure Analysis, Institute of Physics of the Czech Academy of Sciences, Cukrovarnická 112/10, 162 00 Praha 6, Czech Republic

[5] Department of Pharmacy, Faculty of Health and Medical Sciences, University of Copenhagen, Universitetsparken 2, 2100 Copenhagen, Denmark

*Email: miroslav.soos@vscht.cz

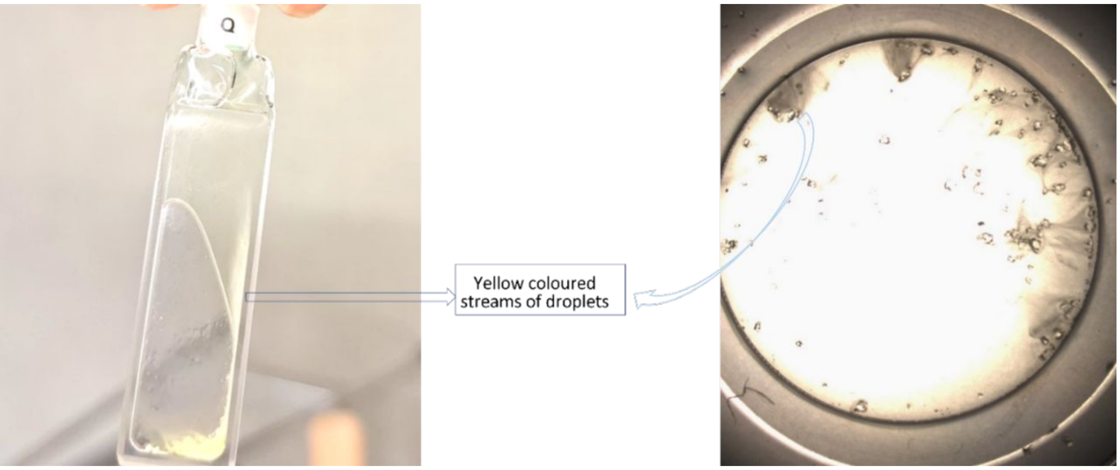

Fig S1 Yellow colored “streams” of droplets

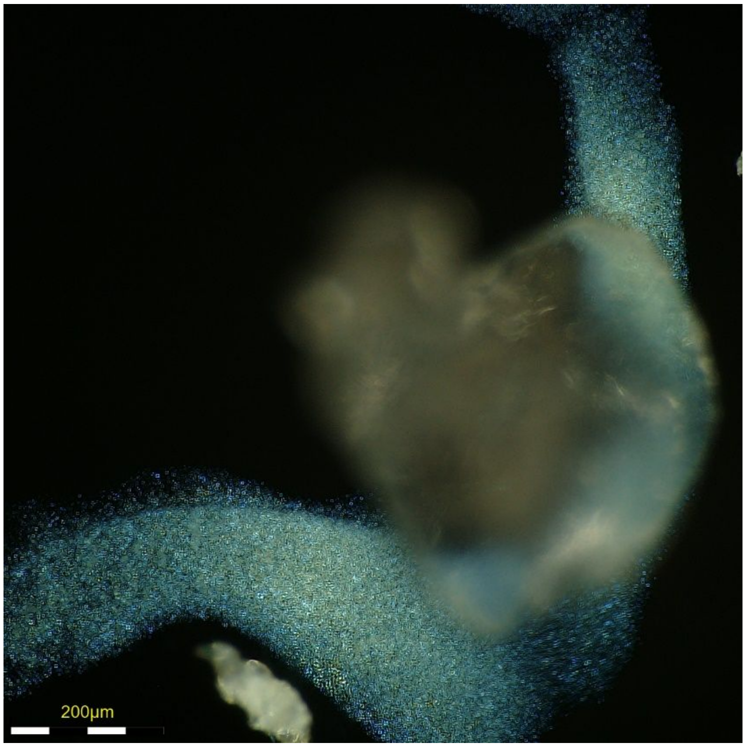

Fig S2 Polarizing light microscopy image of intermediate droplets ‘flowing’ towards an undissolved acid crystal from two sides.

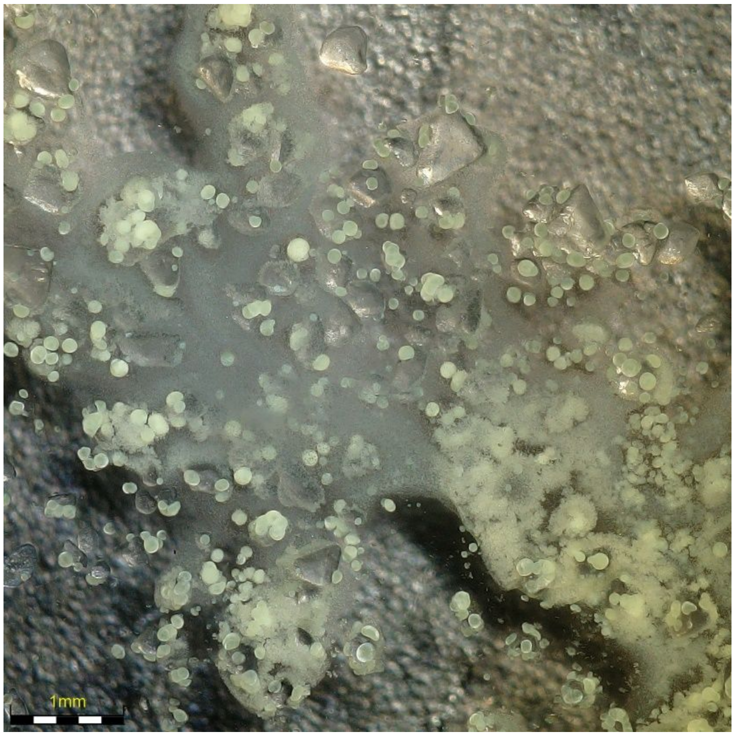

Fig S3 Dark field microscopy image of crystallization pan containing intermediate droplets (off-white 'streams'), acid crystals (transparent) and salt spherulites(yellow) mostly on top of the acid crystals.

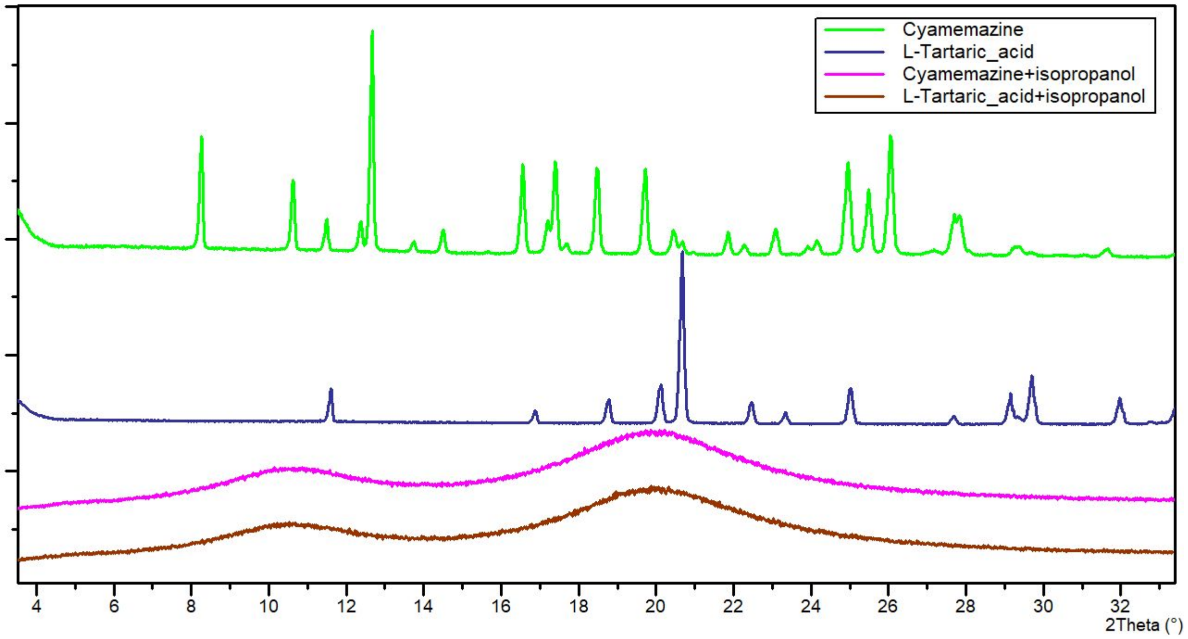

Fig S4 Comparison of XRD patterns of CMZ and L-tartaric acid suspended in isopropanol in capillary to XRPD patterns of solid CMZ and L-tartaric acid crystals

Principal component analysis (PCA) of SRS microscopy images

Fig S5 shows the PCA done on the image (fig. 9(a)) containing cyamemazine crystal and intermediate droplets. PC1 and PC3 are the significant components whereas PC1 and PC4 correspond to baseline and noise respectively. Fig S6 shows the analysis done on the image (fig.10(a) containing the dense phase and salt spherulites. PC1 and PC2 are the significant components in this analysis.

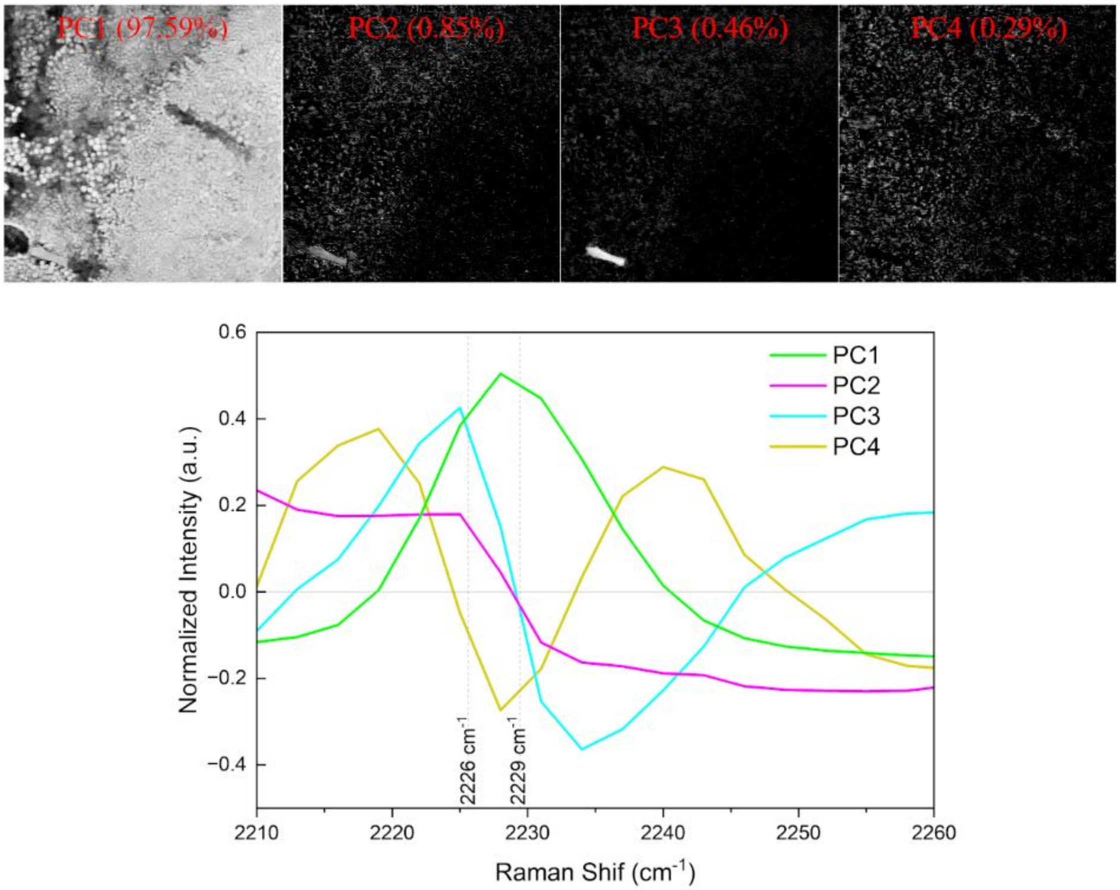

Fig S5 PCA performed on fig. 9 (a)

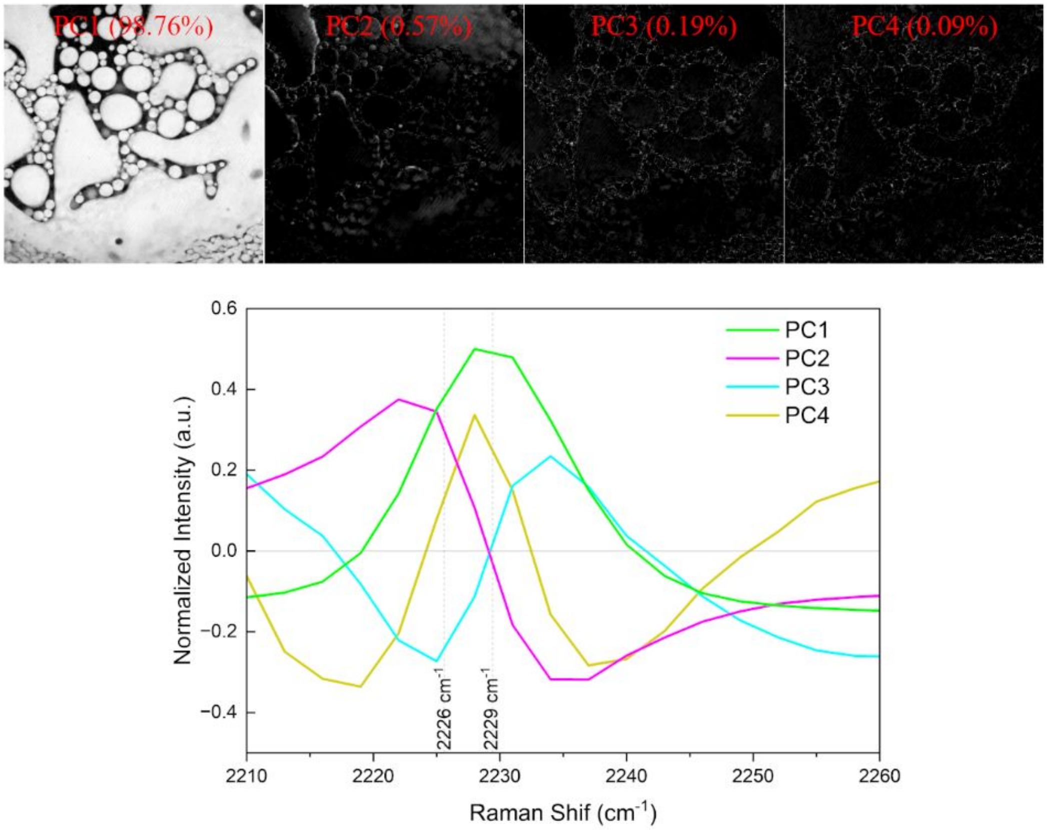

Fig S6 PCA performed on fig. 10 (a)

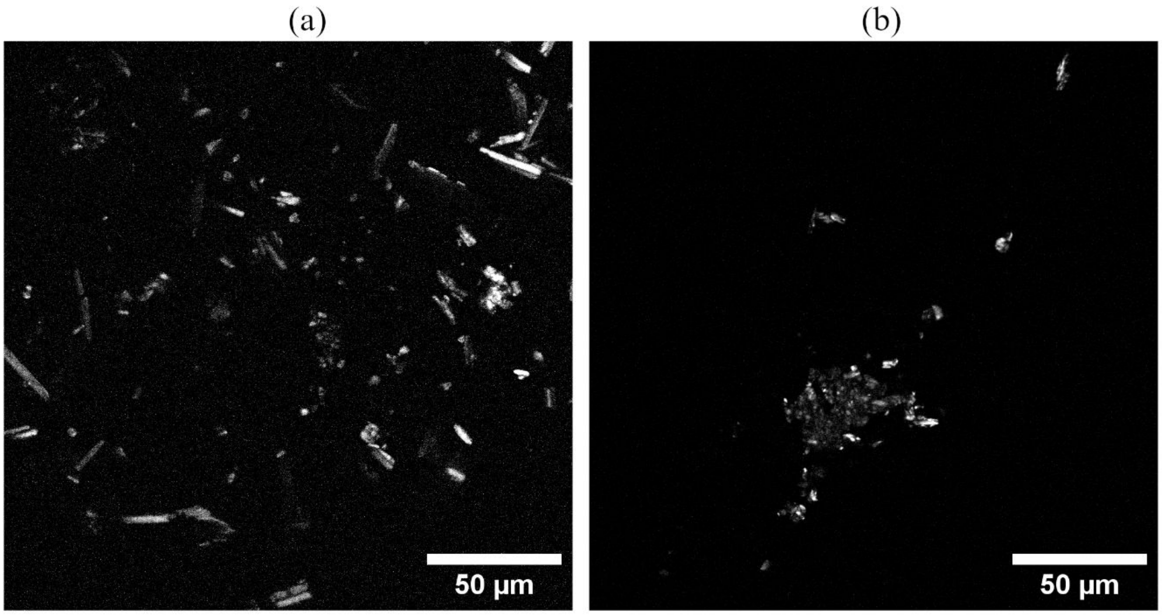

Fig S7 SRS image a) of cyamemazine at 2225 cm^{-1} b) of L-tartrate salt at 2231 cm^{-1}
